# Human adenovirus type 26 uses sialic acid–bearing glycans as a primary cell entry receptor

**DOI:** 10.1126/sciadv.aax3567

**Published:** 2019-09-04

**Authors:** Alexander T. Baker, Rosie M. Mundy, James A. Davies, Pierre J. Rizkallah, Alan L. Parker

**Affiliations:** 1 Division of Cancer and Genetics, School of Medicine, Cardiff University, Heath Park, Cardiff CF14 4XN, UK.; 2 Division of Infection and Immunity, School of Medicine, Cardiff University, Heath Park, Cardiff CF14 4XN, UK.

## Abstract

Adenoviruses are clinically important agents. They cause respiratory distress, gastroenteritis, and epidemic keratoconjunctivitis. As non-enveloped, double-stranded DNA viruses, they are easily manipulated, making them popular vectors for therapeutic applications, including vaccines. Species D adenovirus type 26 (HAdV-D26) is both a cause of EKC and other diseases and a promising vaccine vector. HAdV-D26–derived vaccines are under investigation as protective platforms against HIV, Zika, and respiratory syncytial virus infections and are in phase 3 clinical trials for Ebola. We recently demonstrated that HAdV-D26 does not use CD46 or Desmoglein-2 as entry receptors, while the putative interaction with coxsackie and adenovirus receptor is low affinity and unlikely to represent the primary cell receptor. Here, we establish sialic acid as a primary entry receptor used by HAdV-D26. We demonstrate that removal of cell surface sialic acid inhibits HAdV-D26 infection, and provide a high-resolution crystal structure of HAdV-D26 fiber-knob in complex with sialic acid.

## Teaser

Human adenovirus type 26 uses sialic acid as a primary cellular receptor—structural insights for this phase 3 vaccine vector.

## INTRODUCTION

Adenoviruses are clinically important, both as human pathogens and as platforms for therapeutic applications. As pathogens, human adenoviruses (HAdVs) have been isolated from severe infections in both immunocompromised and healthy individuals (*1*). Some adenoviruses have been associated with acute infection of the eye (*2*), respiratory (*3*), and gastrointestinal tract (*1*). In rare cases, infections prove fatal, as observed in a recent neonatal infection of species D adenovirus type 56 (HAdV-D56) (*4*), among adult patients in New Jersey with HAdV-B7d (*5*) and infamously with HAdV-E4, where large epidemics of adenovirus infection have been seen in military recruits (*6*). However, most fatal infections are observed in immunocompromised individuals (*7*), such as those suffering from graft-versus-host disease (*8*) or HIV (*9*, *10*).

Adenoviruses are classified into seven species (A to G) and between 57 and 90 types depending on the taxonomic definitions used (*11*, *12*). Some adenoviruses have been studied in detail, having well-defined receptor tropisms, including as coxsackie and adenovirus receptor (CAR) (*13*), cluster of differentiation 46 (CD46, also known as membrane cofactor protein) (*14*), desmoglein 2 (DSG2) (*15*), or sialic acid–bearing glycans (*16*). However, most types have low seroprevalence in the population (*17*), though this varies significantly by geographical location (*18*, *19*). Their rarity means that many types remain understudied, with poorly defined primary receptor interactions. This is especially true of the species D adenoviruses (HAdV-D), the largest of the adenoviral species, containing 35 of 57 canonical types (*12*).

Species D adenoviruses are associated with several pathogenicities. HAdV-D56 is a potentially fatal emergent respiratory pathogen composed of a recombination between four species D adenoviruses (*4*). Opportunistic adenovirus infection isolated from patients with HIV/AIDS are most commonly from species D, where they are associated with prolonged shedding in the gastrointestinal tract (*9*). HAdV-D has also been associated with genital disease (*20*). The species D adenoviruses are best known, however, for causing epidemic keratoconjunctivitis (EKC) infections, which is endemic, but not isolated, to Japan (*21*). Classically, the primary EKC causing adenoviruses have been HAdV-D8, HAdV-D37, and HAdV-D64 (*22*) [previously classified as 19a (*23*)]. More recently, other species D adenoviruses have been associated with EKC, including HAdV-D53 (previously classified as HAdV-D22/H8) (*24*, *25*), HAdV-D54 (*26*), and HAdV-D56 (*27*).

The double-stranded adenovirus DNA genome makes them readily amenable to genetic modification (*28*) and therefore has made them attractive candidates for genetic manipulation for therapeutic applications in cancer (oncolytic viruses) (*29*) and as vaccine vectors (*30*). Species D adenoviruses are of special interest as vaccine vectors. Their ability to induce robust cellular and humoral immunogenic responses in humans, coupled with low seroprevalence rates in the general population (*17*), makes them attractive platforms for vaccines, as evidenced by their progression through clinical trials for HIV (*31*), Zika (*32*), and Ebola treatment (*33*). However, there remains a lack of understanding regarding their basic biology and mechanisms of cellular infection. This is exemplified by HAdV-D26, which is being investigated as a vaccine vector for Zika (*32*), HIV (*34*), and respiratory syncytial virus (*35*), and has entered phase 3 clinical trials as an Ebola vaccine (*33*).

Despite its clinical success, recent findings further highlight the lack of clarity over the primary receptor usage of HAdV-D26. It is now clear that, despite previous publications to the contrary, HAdV-D26 cannot engage CD46 as a primary cellular entry receptor (*36*). Instead, the HAdV-D26 fiber knob protein (HAdV-D26K) may engage CAR as a primary receptor, although the affinity of this interaction is attenuated compared to the classical HAdV-C5 interaction with CAR due to the presence of an extended HAdV-D26 fiber knob DG loop, which sterically inhibits the interaction with CAR (*36*). The deduced low affinity of the interaction between CAR and HAdV-D26 fiber knob makes it unlikely that CAR represents the definitive primary receptor of HAdV-D26.

Here, we demonstrate that HAdV-D26 uses sialic acid–bearing glycans as a primary entry receptor and that this interaction can form a productive infection. We solve the structure of HAdV-D26K in complex with sialic acid [*N*-acetylneuraminic acid (Neu5Ac)], revealing a similar topology to the known sialic acid–interacting HAdV-D37 fiber knob in the sialic acid–binding pocket, but highlight crucial mechanistic differences likely to enhance HAdV-D26 affinity for sialic acid compared to other types.

## RESULTS

### HAdV-D26K has an electrostatic profile permissive to sialic acid interaction

Our recent findings highlight that HAdV-D26K is unlikely to use CD46 as a primary cellular receptor. We further confirmed this observation using surface plasmon resonance to confirm that the HAdV-D26 fiber knob protein does not stably interact with either CD46 or DSG2. A weak affinity for CAR was observed for HAdV-D26K; however, this is unlikely to represent a primary receptor. Previous amino acid sequence alignments demonstrated little conservation of sialic acid–binding residues with the fiber knob domains of the known sialic acid using HAdV-G52SFK (short fiber knob) or canine adenovirus type-2 (CAV-2) (*36*). However, these alignments indicated that HAdV-D37, known to bind sialic acid in the apex of the fiber knob, bore some similarity at a sequence level. We sought to evaluate the ability of HAdV-D26K to interact with the remaining previously described adenovirus receptor, sialic acid.

**Fig. 1:**
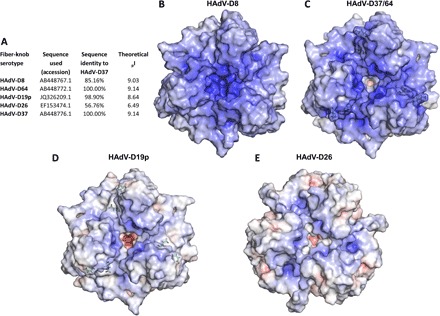
HAdV-D26K forms a local basic area in the apical depression to facilitate sialic acid binding despite an overall acidic predicted isoelectric point. (**A**) HAdV-D26K has low (56.76%) sequence identity with fiber knobs known to bind sialic acid by a similar mechanism and an acidic isoelectric point. The electrostatic potential surfaces of HAdV-D8K (**B**), HAdV-D64/37 (**C**), and HAdV-D19p (**D**) fiber knobs are highly basic, especially about the central depression about the threefold axis. (**E**) HAdV-D26 fiber knob is less basic overall but maintains positive potential in the central depression. Surfaces are displayed at ±10 mV, and the two residues that differ between HAdV-D19p and HAdV-D37/64 are shown as green sticks.

HAdV-D37 fiber knob is identical to that of HAdV-D64 and highly homologous to that of HAdV-D8 (Fig. 1A). These three viruses have been shown to cause EKC and to interact with sialic acid. The closely related HAdV-D19p, differing from HAdV-D64 at only two residues, has also been shown to bind sialic acid, but does not cause EKC. We compared HAdV-D26K to these viruses to determine whether a similar binding mechanism was possible.

These sialic acid–binding viruses all have highly negative predicted isoelectric points (pIs) (Fig. 1A). We calculated the surface electrostatic potentials of these fiber knob proteins at pH 7.35 to simulate the pH of extracellular fluid, using previously published crystal structures where available. There is no published structure of HAdV-D8K; thus, we generated a homology model based on the closest known relative with a crystal structure (Fig. 1B).

The analyzed fiber knob proteins are highly charged. We observed a concentration of positive charge in the central depression around the threefold axis, which corresponds to the previously reported sialic acid–binding sites (Fig. 1, B to D). HAdV-D8 has the most basic surface potential (Fig. 1B), followed by HAdV-D37/64 (Fig. 1C). HAdV-D19p is less basic due to the two amino acid substitutions, compared to HAdV-D37/64, though the central depression is unaffected, as has previously been noted (Fig. 1D) (*37*).

HAdV-D26K has a lower predicted pI, 6.49, and a less positive surface potential (Fig. 1, A and E). However, the central depression of HAdV-D26K remains basic around the region where sialic acid is observed to bind in HAdV-D19p and HAdV-D37. HAdV-D26 retains the charge needed for sialic acid binding in the apex of the protein in the context of an otherwise acidic protein (Fig. 1E).

### HAdV-D26 requires cell surface sialic acid for efficient infection

**Fig. 2:**
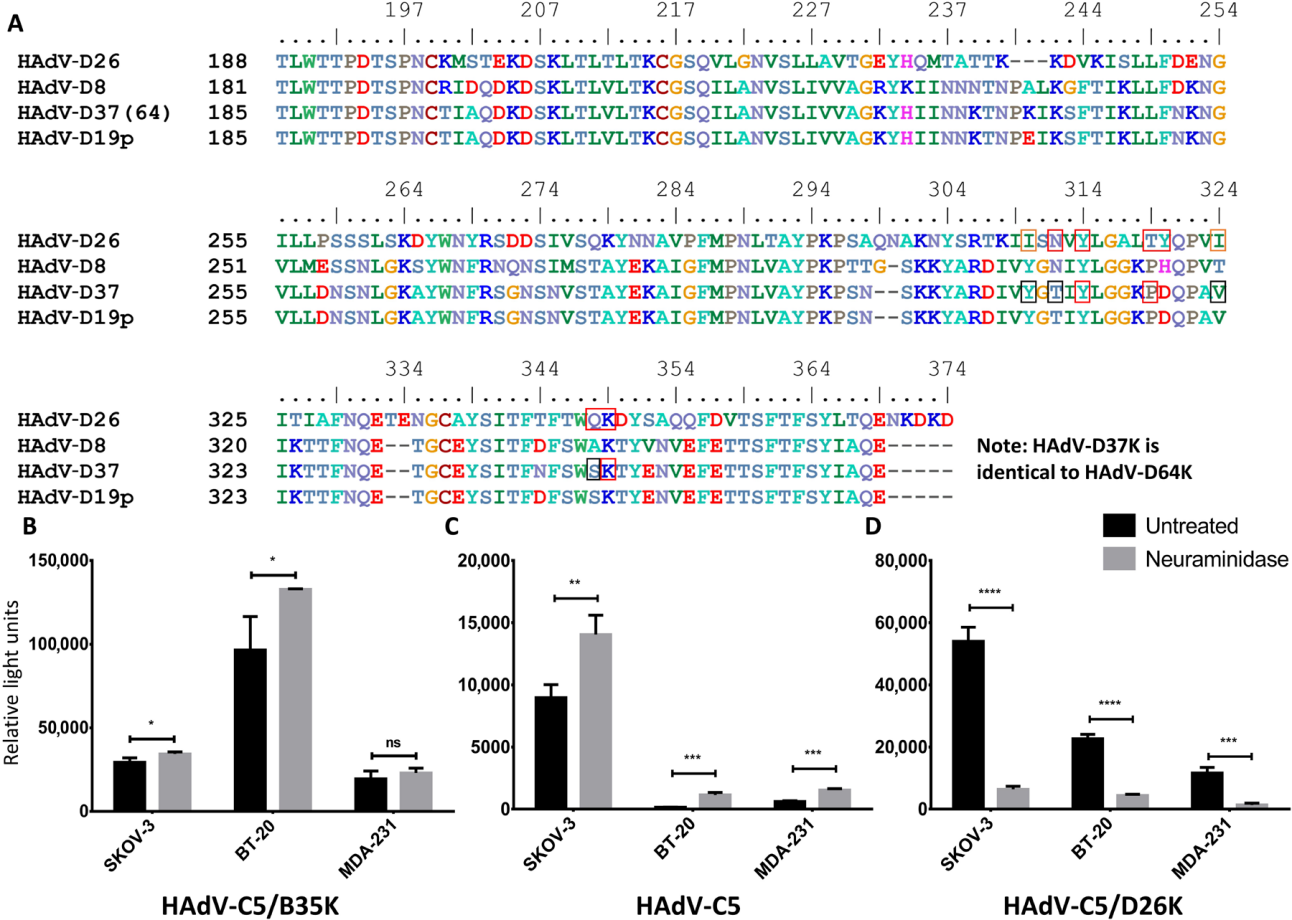
HAdV-D26K shares key binding residues with sialic acid–using adenoviruses and exploits sialic acid to infect cells. (**A**) Sequence alignment of HAdV-D26K shows conservation of key binding residues with known sialic acid–binding adenoviruses; top numbering is according to HAdV-D26K. Residues boxed in red form polar contacts with sialic acid, those boxed in black denote contact sialic acid via water bridge, and those boxed in orange indicate hydrophobic contacts; all HAdV-D26K polar contacts also form water bridges. Neuraminidase treatment does not reduce the ability of HAdV-D5/B35K (**B**) or HAdV-C5 (**C**) to infect SKOV-3 (ovarian adenocarcinoma), BT-20 (breast carcinoma), or MDA-231 (metastatic breast adenocarcinoma) cells, while HAdV-D5/D26K (**D**) is significantly inhibited. *n* = 3 biological replicates; error bars indicate ±SD.

Sequence alignment of HAdV-D26K with these known sialic acid–using viruses, bearing a positively charged apex, showed conservation of key binding residues between types (Fig. 2A). We observe complete conservation of Tyr^314^ and Lys^349^ across the four types and conservation of Asp^312^ with HAdV-D8 (Fig. 2A). Furthermore, while HAdV-D26K Tyr^320^ is not conserved, inspection of the crystal structure of HAdV-D37K and HAdV-D19p in complex with sialic acid [Protein Data Bank (PDB) 1UXA and 1UXB, respectively] (*37*) reveals this to be a main-chain oxygen contact, positioned similarly in HAdV-D26, and can be considered homologous.

To investigate the ability of HAdV-D26 to use sialic acid as a cell entry receptor, we used a replication-incompetent HAdV-C5 vector pseudotyped with the HAdV-D26 fiber knob, expressing a green fluorescent protein (GFP) transgene. We performed infectivity studies in three cell lines, with and without pretreatment with neuraminidase, to remove cell surface sialic acid. The tested cell lines could be infected by the CD46-mediated (Fig. 2B) or CAR-mediated (Fig. 2C) pathways to some extent, by HAdV-C5/B35K or HAdV-C5, respectively. However, infection via these routes was uninhibited by neuraminidase treatment. Transduction efficiency of HAdV-C5 and HAV-C5/B35K was actually enhanced by neuraminidase treatment in some cases, an effect that has been previously observed (*38*, *39*). This has been suggested to be due to a reduction in the electrostatic repulsion of the negatively charged capsid of HAdV-C5.

Infection by the HAdV-C5/D26K pseudotype was significantly reduced in all three cell lines following treatment with neuraminidase (Fig. 2D). This inhibition is significant (*P* < 0.005), resulting in >5-fold decrease in infection in all three cell lines tested, similar to the decrease observed when performing similar experiments with EKC-causing viruses HAdV-D37/53/64, of five- to eightfold inhibition (*40*). These data indicate that HAdV-C5/D26K is using sialic acid, not CD46 or CAR, to infect these cells.

### HAdV-D26 forms a stable complex with sialic acid

**Fig. 3:**
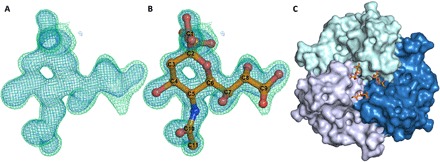
Sialic acid binds in the apical depression of HAdV-D26 fiber knob protein. The map shows clear density for a ligand (**A**), which is best described by a double conformer of sialic acid (**B**). (**C**) Sialic acid (orange) is seen to bind in three locations in the apical depression of the HAdV-D26 fiber knob, bridging between monomers (shades of blue) of the trimeric assembly. Crystallization statistics are provided in table S1; 2*F*_o_*F*_c_ map (blue mesh, σ = 1.5) and *F*_o_*F*_c_ (green mesh, σ = 3.0).

We crystallized HAdV-D26K in complex with sialic acid to clarify the mechanism of interaction. Refinement of structures generated from HAdV-D26K crystals soaked in sialic acid shows electron density for a small-molecule ligand in the apical depression (Fig. 3A); this is best described by a racemic mixture of α and β anomers, in conjunction with double conformations of sialic acid (Fig. 3B). The cubic space group (table S1) enabled assembly of the biological trimer. We observed three copies of sialic acid bound within the apex of the fiber knob trimer (Fig. 3C), as previously observed in HAdV-D37 and HAdV-D19p.

Sialic acid binding was observed in structures crystallized at both pH 8.0 (PDB 6QU6) and pH 4.0 (PDB 6QU8). Observation of sialic acid density at high σ values suggests a highly stable interaction (fig. S1). Electron density demonstrates the C2 carboxyl and OH groups in two conformations, and the C6 glycerol group is flexible, with the C7-C8 bond rotating to alter the orientation of the glycerol arm, relative to the pyranose ring and binding pocket (Fig. 3, A and B, and fig. S1). The glycerol group exhibits further flexibility at the C8-C9 bond, making the terminal oxygen mobile. The distribution of the density for the glycerol group is different at each pH (fig. S1), suggesting that pH could affect the preferred mode of interaction.

The most biologically relevant sialic acid conformation places the carboxyl group axial to the chair conformation pyranose ring (fig. S2), leaving the OH group pointing away from the fiber knob and free to form an α(2)-glycosidic bond as part of a glycan. This is suggestive of a terminal sialic acid residue, as the chain can extend out of the central depression, as was observed in the previously described HAdV-D37K:GD1a glycan structure (*37*, *41*).

### HAdV-D26 has a sophisticated sialic acid–binding pocket

**Fig. 4:**
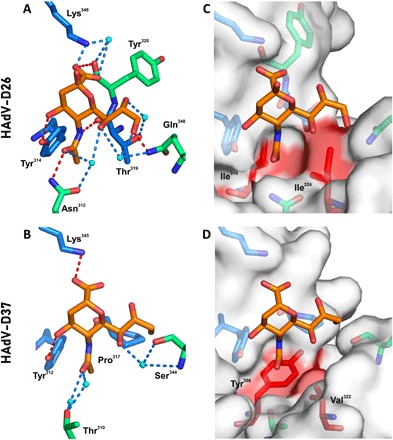
HAdV-D26K forms a complex interaction network of hydrophobic and electrostatic interactions with sialic acid. Sialic acid (orange) is seen to bind HAdV-D26 (**A**) and HAdV-D37 (**B**) through a network of polar contacts (red dashes) and hydrogen bonds (blue dashes). The interaction is stabilized by hydrophobic interactions (red regions on white surface) with the *N*-acetyl CH_3_ group, but different residues in HAdV-D26 (**C**) and HAdV-D37 (**D**). Waters are shown as cyan spheres, residues forming comparable contacts in HAdV-D26 and HAdV-D37 are shown as blue sticks, and other residues are shown as green sticks. Oxygen and nitrogen are seen in red and blue, respectively.

Comparison between the HAdV-D26K and HAdV-D37K, the best described of the sialic acid–binding adenoviruses, reveals that several sialic acid contacts are conserved (Fig. 4, A and B). Lys^349^ and Tyr^314^ are identical, and while Lys^349^ exhibits some flexibility, all observed lysine conformations form a contact with the carboxyl group of the sialic acid (fig. S3). While Thr^319^ is not conserved in HAdV-D37 (which has a proline at this position), the main-chain oxygen contact to the *N*-acetyl nitrogen is spatially similar; hence, the bond can be considered homologous.

The HAdV-D26K sialic acid interface forms further contacts with sialic acid that are not observed in HAdV-D37K (Fig. 4, A and B). HAdV-D26K contacts the *N*-acetyl oxygen of sialic acid using Asn^312^, which forms a polar contact and a water bridge (Fig. 4A). The comparable residue in HAdV-D37K, Thr^310^, is too short to form a direct polar interaction (Fig. 4B); instead, a pair of water bridges is used.

In HAdV-D37, the glycerol arm of sialic acid was only contacted by a water bridge between Ser^344^ and the C7-OH. However, in HAdV-D26, all three OH groups in the glycerol arm form contacts. C7-OH is coordinated by water bridges to both Asn^312^ and Gln^348^. C8-OH forms a water bridge with Thr^319^, and C9-OH forms both a water bridge and a polar contact directly to Gln^348^. Similar to Thr^310^, the serine belonging to HAdV-D37 at position 344 is too short to form a polar bond equivalent to the one with Gln^348^.

Notably, the density for the glycerol arm of sialic acid suggests several possible conformations (fig. S2), which can be interpreted as flexibility. However, we suggest that, in HAdV-D26, this is unlikely since it is so well coordinated in all conformations observed, at both pH 8.0 and pH 4.0 (fig. S3). We propose that HAdV-D26K can form a stable interaction with the glycerol arm, regardless of the specific confirmation. The variable density can be explained as the average distribution (or partition) of the different discrete positions.

We also observe a hydrophobic interaction in HAdV-D26 with the *N*-acetyl methyl group at C11 (Fig. 4C). A similar hydrophobic interaction is seen in HAdV-D37, where Tyr^312^ and Val^322^ form a hydrophobic patch (Fig. 4D), but the HAdV-D26 interaction appears to be more selective, where Ile^310^ and Ile^324^ form a hydrophobic cradle around the methyl group (Fig. 4C).

### HAdV-D26 binds sialic acid through an induced-fit mechanism

**Fig. 5:**
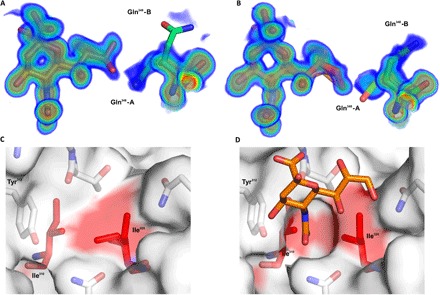
HAdV-D26K uses an induced-fit mechanism in sialic acid binding. HAdV-D26K residue Gln^348^ can occupy multiple conformations, with a greater preference for conformation A (capable of forming a polar contact with the glycerol arm of sialic acid) at pH 8.0 (**A**) than at pH 4.0 (**B**). (**C**) Ile^324^ has two conformations when HAdV-D26K is unliganded (PDB 6FJO). (**D**) However, upon sialic acid binding, the Ile^324^ adopts a single confirmation, creating a hydrophobic indentation around the *N*-acetyl methyl group bounded by Ile^324^, Ile^310^, and the ring of Tyr^312^.

We observe split density for Gln^348^ in both pH 8.0 (Fig. 5A) and pH 4.0 (Fig. 5B). While conformation A can form polar contacts with sialic acid, conformation B points into the solvent and cannot. It is possible that Gln^348^ is flexible, but then is attracted to the charged density of the glycerol arm upon sialic acid interaction. We also observe greater occupancy of conformation A in the pH 8.0 structure (approximately 0.7), while at pH 4.0, the occupancy is evenly split. This suggests that the interaction may be more stable at higher pH, such as that associated with the pH found at the cell surface.

Ile^324^, which is seen to be involved in hydrophobic interactions with the *N*-acetyl methyl group (Fig. 4C), can also have multiple conformations. In an unliganded structure of HAdV-D26 fiber knob (PDB 6FJO), the long arm of Ile^324^ is seen to rotate (Fig. 5C). However, in the ligated structure, Ile^324^ occupies a single conformation (Fig. 5D), forming a cradle. This creates a larger hydrophobic patch and restricts the methyl group in space by pinching it between the pair of hydrophobic isoleucines, anchoring the *N*-acetyl group.

## DISCUSSION

Other adenoviruses have been shown to interact with sialic acid. These include CAV-2 (*42*), Turkey adenovirus 3 (*43*), and HAdV-G52 short fiber knob (*44*, *45*), but these viruses interact with sialic acid in lateral regions of the fiber knob, dissimilar from HAdV-D26K. Four other HAdV fiber knob proteins (HAdV-D8/19p/37/64K) have been previously shown to use sialic acid, binding in the apical region. These viruses have high sequence similarity to each other, but not to HAdV-D26K, though they all share key sialic acid contact residues (Figs. 1A and 2A).

The structure of HAdV-D8 has not been determined, either alone or in complex with sialic acid, but infection by HAdV-D8 is sensitive to neuraminidase treatment, suggesting sialic acid utilization (*46*). Furthermore, HAdV-D8K has very high sequence homology and shared sialic acid contact residues with HAdV-D19p/37K, making it logical to expect a similar interaction mechanism. In support of this, we observe a similar electrostatic profile in the modeled fiber knob as seen in HAdV-D37/64 (Fig. 1, B and C). HAdV-D64 has an identical fiber knob domain to that of HAdV-D37; thus, fiber knob interactions with sialic acid are likely to be conserved between these types. HAdV-D26K conserves the key region of positive potential in the apical depression, but in the context of an otherwise more acidic protein (Fig. 1E).

Inspection of the sialic acid–binding pocket of HAdV-D26K reveals a much more complex mechanism of interaction than that previously reported for HAdV-D37K (Fig. 4) (*37*). The overall topology of the pocket is similar, with hydrophobic residues around the *N*-acetyl group and polar contacts between the carboxyl and C4-OH group. However, HAdV-D26K has several differences that increase the number of contacts between the sialic acid and the fiber knob.

Subtle sequence changes enable more numerous interactions between HAdV-D26K and sialic acid than are possible in HAdV-D37K. In HAdV-D37, Pro^317^ forms a main-chain oxygen contact to the nitrogen of sialic acid; however, it also creates tension, which rotates the N-terminal residue away from the carboxyl group of sialic acid. In HAdV-D26K, Tyr^320^, which is C-terminal of the Thr^319^ that is equivalent to Pro^317^ in HAdV-D37K, does not create this tension and enables the main-chain oxygen at position 320 to contact the sialic acid carboxyl group. Thr^319^ also forms a water bridge with the C8-OH group, helping to stabilize the glycerol side chain (Fig. 4A).

This is one of several examples of HAdV-D26K forming additional contacts with sialic acid that are not observed with HAdV-D37/19p. The substitution of the Thr^310^ and Ser^344^ found in HAdV-D37K for longer charged residues (Asn^312^ and Gln^348^, respectively) in HAdV-D26K enables direct polar contacts, as well as additional water-bridge contacts. Substitution of Tyr^308^ and Val^322^ for more hydrophobic isoleucine residues in HAdV-D26 (Ile ^310^ and Ile^324^, respectively) creates a hydrophobic indentation better tailored to fit around the *N*-acetyl methyl group.

The high resolution of the datasets generated to determine the sialic acid–bound HAdV-D26K structure enables visualization of multiple residue conformations with partial occupancy. In unliganded structures of HAdV-D26K (PDB 6FJO), Ile^324^ exhibits a double conformer, occupying the available space (Fig. 5C). However, when sialic acid is bound, it is restricted to have a single conformation with the long arm facing away from the sialic acid site, toward the intermonomer cleft. Ile^310^ has the opposite orientation and creates an indentation, which cradles the methyl group of sialic acid. Tyr^312^ may further contribute to the hydrophobic cradle. Tyr^312^ would not normally be considered a hydrophobic residue, but the side-chain oxygen faces toward the solvent, where it forms a polar interaction with the C4-OH on sialic acid (Fig. 4A), leaving the face of the tyrosine ring exposed to the methyl group, which may contribute hydrophobic character to the cradle. This tyrosine behaves in both a polar and hydrophobic manner at the same time. We suggest that the long arm of Ile^324^ adopts the sialic acid–binding conformation in response to the hydrophobic pressure exerted by sialic acid entering the pocket, minimizing the exposed hydrophobic surface when unbound, holding the methyl group between the short arm of Ile^310^ and the Tyr^312^ ring, making this an example of induced fit.

The double occupancy of Gln^348^ may indicate a second induced-fit mechanism. We observe two possible conformations of Gln^348^ (Fig. 5C). While conformation A does not form any contacts, conformation B forms a polar bond and water bridge, with the sialic acid glycerol group. In HAdV-D37K, the glycerol group forms only a water bridge from C7-OH to Ser^344^, the spatial equivalent of Gln^348^. While we have not determined the preferred conformation of the sialic acid glycerol group versus Gln^348^ conformation, we observed that it can form polar contacts with it regardless, while Gln348 is in conformation B (Fig. 5C).

We suggest that Gln^348^ may be labile until the binding of sialic acid. Upon sialic acid binding, Gln^348^ becomes attracted to the charged glycerol group, causing it to stabilize in conformation A. This has the effect of “locking” the glycerol side chain in place, which is further restrained by water-bridge contacts to Thr^319^ and Asn^312^.

Gln^348^ has greater occupancy in a sialic acid–binding conformation (conformation A) at pH 8.0, which corresponds more closely to the physiological conditions in which it would encounter at the cell surface (Fig. 5A). At pH 4.0, Gln^348^ has approximately half occupancy in each conformation (Fig. 5B). This implies the possibility of HAdV-D26K having lower sialic acid affinity under more acidic conditions, such as those encountered during endosomal trafficking down the lysosomal pathway.

Therefore, the HAdV-D26K binding pocket to sialic acid is summarized by three synchronous mechanisms: an *N*-acetyl anchor composed of a polar contact to Asn^312^ stabilized by a water bridge and an induced hydrophobic cradle around the methyl group; an inducible lock, where Gln^348^ forms a polar contact to the most terminal atoms in the glycerol arm, supported by a network of water bridges; and, last, a network of polar contacts to the carboxyl, C4 oxygen, and nitrogen atoms, which stabilize the pyranose ring.

This interaction in HAdV-D26K is a much more sophisticated binding mechanism compared to HAdV-D19p and HAdV-D37/64. However, the overall pocket topology and several key residues bear similarities. It may be unexpected to observe such similarity given the low level of sequence homology HAdV-D26K has to the HAdV-D37K (56.76%, Fig. 1A). Other regions, especially the loops, have highly dissimilar sequences. There is precedent for this within adenovirus, with recombination events being reported in numerous settings (*23*, *24*).

It has previously been suggested that many of the species D adenoviruses may have dual sialic acid–binding affinity and CAR affinity (*37*). This has been observed in HAdV-D37/64, CAV-2 (*13*, *42*), and, now, HAdV-D26 (*36*). The species G adenovirus HAdV-G52 has also been observed to bind both CAR and sialic acid, but using two different fiber knob proteins on the same virus and a different mechanism of sialic acid interaction in the knob (*44*), which is shown to bind polysialic acid (*47*). Previous work has proposed that CAR may be a receptor for many, if not all, of the species D adenoviruses with variable affinity (*36*, *37*) and suggests that sialic acid could also be widely used (*37*). These findings support that assertion, adding another species D adenovirus, with low sequence similarity, to the pool of adenoviruses observed to bind both CAR and sialic acid.

HAdV-D43, HAdV-D27, and HAdV-D28 fiber knobs share high sequence homology with HAdV-D26K, sharing most of the critical binding residues, and/or having structural homologs at those positions (fig. S4). HAdV-D26K is the only species D adenovirus to have a glutamine at position 348 (HAdV-D26K numbering), though many have the shorter (but similarly charged) asparagine at this location, share the serine or similarly charged residue found in HAdV-D37K, or have an asparagine that could behave similarly to glutamine. However, HAdV-D8 has an uncharged alanine at this position, suggesting that a charged residue may not be strictly required for sialic acid binding, though it may alter affinity (fig. S4).

HAdV-D8K shares an asparagine at the same position as HAdV-D26K, which we have shown to form polar and water-bridge contacts to sialic acid (Fig. 4A). While this is unique among the classical EKC-causing viruses HAdV-D19p/37/64, it is the most common residue at this position in the species D adenoviruses (fig. S4).

The HAdV-D26K surface electrostatics most closely resemble those of HAdV-D19pK. HAdV-D19pK is capable of binding sialic acid (*37*), and a limited effect is seen on infection of A549 cell binding after neuraminidase treatment to remove cell surface sialic acid (*46*). HAdV-D19p binding to Chang C (human conjunctival) cells was completely unaffected by neuraminidase treatment, though binding was very low regardless of neuraminidase treatment (*38*). This inability to bind Chang C cells was shown to depend on a single lysine residue (Lys^240^) in the apex of the fiber knob, but distant from the sialic acid–binding pocket, creating a more acidic apical region in the lysine’s absence (*48*). HAdV-D26K also lacks a lysine in this position and has the most acidic electrostatic profile observed in this study (Fig. 1).

HAdV-D37K and the identical HAdV-D64K have been shown to preferentially interact with the sialic acid–bearing GD1a glycan on the corneal cell surface, causing EKC (*41*). However, it seems unlikely that a protein capable of trivalent sialic acid binding is completely specific for GD1a, a disialylated glycan, given the wide range of available glycan motifs that are di- and trisialylated. The GD1a preference may be diminished in HAdV-D19p by the acidic surface caused by the two amino acid substitutions, creating a glycan preference for tissues outside of the eye. Similarly, HAdV-D26K may have a unique glycan preference, driving its tissue tropism toward cells with different glycosylation patterns.

The infectivity assays demonstrate sialic acid utilization by HAdV-C5/D26K in multiple cell types (Fig. 2D), suggesting either that HAdV-D26K binds to a glycosylation pattern conserved in all three cell lines or that HAdV-D26K can bind to variable glycosylation motifs. In this assay, a non–sialic acid–using HAdV-C5 virus (Fig. 2C) pseudotyped with the fiber knob domain of HAdV-D26 was used. This pseudotype (and HAdV-C5/B35K) retains the fiber shaft domain of HAdV-C5, which is 212 amino acids longer than the native HAdV-D26 fiber shaft. This could potentially increase the avidity of the virus for its receptor, providing greater range and flexibility than the equivalent short species D fiber shaft. However, it is unlikely to affect the receptor binding that is largely governed by the interaction with the knob portion of the fiber protein.

Previous work by Nestić *et al*. (*49*) suggested that ανβ3 integrin is used by HAdV-D26 during infection and provided evidence for a role for CAR as a low-affinity receptor for HAdV-D26, supporting our previous structural and biological observations (*36*). Enhanced αvβ3-dependent infection by HAdV-D26 is likely explained by secondary interactions with the penton base, which has a shorter Arginine, Glycine, Aspartic Acid (RGD)–containing loop than HAdV-C5, demonstrated previously using cryo–electron microscopy (*50*). The infectivity of the pseudotyped HAdV-C5/D26K vector described here is not limited by this reduced integrin affinity due to the retention of the HAdV-C5 penton base protein; thus, conclusions cannot be drawn from the present study regarding secondary interactions that govern internalization of HAdV-D26.

Our findings clarify the receptor tropism of HAdV-D26 and build upon the increasingly complex body of knowledge describing species D adenoviruses. The comparison of different sialic acid–binding residues suggests greater plasticity regarding the specific residues needed for sialic acid binding than previously thought (fig. S4). It seems highly likely that many adenoviruses in species D and perhaps other species may interact with sialic acid in this manner. This suggests potential causes of off-target infection by species D–derived viral vectors. Conversely, investigation of their specific glycan preferences may enable more tissue-specific targeting. Knowledge of the sialic acid–binding mechanism suggests mutations, which may ablate sialic acid interaction, enabling engineering of better restricted tropisms for future virotherapies. This knowledge regarding HAdV-D26 receptor can inform clinical practice in the rare cases of acute HAdV-D26 infection or in the face of adverse reactions to HAdV-D26–based vaccines, suggesting that sialic acid–binding inhibitors, such as Zanamivir, or trivalent sialic acid derivatives (*51*) may make effective anti–HAdV-D26 therapies.

## MATERIALS AND METHODS

### Generation of fiber knob pseudotyped HAdV-C5 viral vectors

HAdV-C5 viruses pseudotyped with the HAdV-D26 or HAdV-D35 fiber knob proteins were generated by the recombineering method, as published by Stanton *et al*. (*28*). In brief, a marker cassette was generated using the SacB cassette template (*28*), with the SacB primer pair (table S1) with homology to the HAdV-C5 fiber knob DNA sequence before the Threonine, Leucine, Tryptophan (TLW) hinge sequence (forward primer) and after the stop codon (reverse primer). This template was integrated into a bacterial artificial chromosome (BAC) containing the genome of a GFP-expressing HAdV-C5, which had been rendered replication incompetent by deletion of the E1A gene, now containing a marker cassette instead of the HAdV-C5 fiber knob domain.

The DNA sequence of the HAdV-D26K and HAdV-B35K fiber knobs was amplified using the 26K and 35K primer pairs, respectively (table S1), containing similar homology to the SacB cassette. A second round of recombineering was used to generate the final HAdV-C5 pseudotyped genomes by integrating the HAdV-D26K and HAdV-B35K polymerase chain reaction (PCR) transcripts. After recombineering, the new BACs were sequenced to confirm the correct fiber knob DNA sequence.

The BAC DNA for the new vector genomes was transfected into 293 cell line stably expressing the tetracycline repressor protein (TREx-293) cells (1.5 × 10^6^), cultured in a T25 cell bind flask (Corning) in 5 ml of Dulbecco’s modified Eagle’s medium (DMEM, Gibco) supplemented with 10% v/v fetal bovine serum, using the effectene system (QIAGEN). Cells were kept in culture until a cytopathic effect (CPE) was observed, at which point they were harvested by scraping and centrifugation at 1200*g* for 3 min. Cells were resuspended in 1 ml of media and frozen at −80°C to create a crude stock of virus. TREx-293 cells were cultured at 70% confluency in 5× T150 cell bind flasks (Corning) containing 20 ml of complete DMEM and then inoculated with 10 μl of crude virus stock. Cells were maintained in culture until CPE was observed and harvested by scraping and centrifugation at 1200*g*.

Virus was then purified from this cell pellet using the Cesium Chloride (CsCl) gradient method (*52*). Virus titer was determined in viral particles per milliliter (VP/ml) using the Pierce BCA Protein Assay Kit, assuming 4 × 10^9^ VP/μg of protein (*52*). By using this method, we were able to generate HAdV-C5 viruses pseudotyped with the fiber knob domains of HAdV-D26 or HAdV-B35. These viruses retained the HAdV-C5 fiber shaft domain and were replication incompetent.

### Infectivity assays

Cells were seeded at a density of 30,000 cells per well in a flat-bottomed 96-well cell culture plate and incubated overnight at 37°C to adhere. Cells were washed twice with 200 μl of phosphate buffered saline (PBS), and 50 μl of neuraminidase (Sigma-Aldrich, cat. no. 11080725001) was added to the appropriate wells at a concentration of 50 mU/ml, diluted in serum-free media, and incubated for 1 hour at 37°C. Cells were cooled on ice and washed twice with 200 μl of PBS. GFP-expressing, replication-incompetent viruses were added to the appropriate wells at a concentration of 2000 or 5000 VP per cell, in 100 μl of serum-free media, at 4°C, and incubated on ice for 1 hour. Serum-free media alone were added to uninfected control wells. Cells were washed twice with 200 μl of cold PBS, and complete media were added (DMEM, 10% fetal calf serum) and incubated for 48 hours at 37°C. Cells were then trypsinized and transferred to a 96-well V-bottom plate, washed twice in 200 μl of PBS and fixed in 2% paraformaldehyde for 20 min before wash, and resuspended in 200 μl of PBS.

Samples were run in triplicate and analyzed by flow cytometry on Attune NxT (Thermo Fisher Scientific), analyzed using FlowJo v10 (FlowJo, LLC), gating sequentially on singlets, cell population, and GFP-positive cells. Levels of infection were described in terms of total fluorescence, defined as the percentage of GFP-positive cells (% + percentage positive) multiplied by the median fluorescence intensity of the GFP-positive population.

### Amino acid sequence alignments

Representative whole genomes of HAdV-D64, HAdV-D19p, HAdV-D26, and HAdV-D37 were selected from the National Center for Biotechnology Information (NCBI), and the fiber knob domain amino acid sequences were derived from them, defined as the translated nucleotide sequence of the fiber protein (pIV) from the conserved TLW hinge motif to the protein C terminus. The fiber knob domains were aligned using the European Bioinformatics Institute Clustal Omega tool (*53*).

### Generation of recombinant fiber knob protein

SG13009 *Escherichia coli* harboring pREP-4 plasmid and pQE-30 expression vector containing the fiber knob DNA sequence were cultured in 20 ml of LB broth with ampicillin (100 μg/ml) and kanamycin (50 μg/ml) overnight from glycerol stocks made in previous studies (*54*). One liter of Terrific Broth (modified, Sigma-Aldrich) containing ampicillin (100 μg/ml) and kanamycin (50 μg/ml) was inoculated with the overnight *E. coli* culture and incubated at 37°C until they reached an optical density of 0.6. Isopropyl-β-d-thiogalactopyranoside was then added to a final concentration of 0.5 mM, and the culture was incubated at 37°C for 4 hours. Cells were then harvested by centrifugation at 3000*g*, resuspended in lysis buffer [50 mM tris (pH 8.0), 300 mM NaCl, 1% (v/v) NP-40, lysozyme (1 mg/ml), and 1 mM β-mercaptoethanol], and incubated at room temperature for 30 min. Lysate was clarified by centrifugation at 30,000*g* for 30 min and filtered through a 0.22-μm syringe filter (Millipore, Abingdon, UK). Clarified lysate was then loaded onto a 5-ml HisTrap FF nickel affinity chromatography column (GE Healthcare) at 2.0 ml/min and washed with 5 column volumes into elution buffer A [50 mM tris (pH 8.0), 300 mM NaCl, and 1 mM β-mercaptoethanol]. Protein was eluted by 30-min gradient elution from buffer A to B (buffer A + 400 mM imidazole). Fractions were analyzed by reducing SDS–polyacrylamide gel electrophoresis (SDS-PAGE), and fiber knob-containing fractions were further purified using a Superdex 200 10/300 size exclusion chromatography column (GE) in crystallization buffer [10 mM tris (pH 8.0) and 30 mM NaCl]. Fractions were analyzed by SDS-PAGE and pure fractions were concentrated by centrifugation in VivaSpin (10,000 molecular weight cutoff) (Sartorius, Goettingen, Germany) preceding crystallization.

### Crystallization and structure determination

Protein samples were purified into crystallization buffer [10 mM tris (pH 8.0) and 30 mM NaCl]. The final protein concentration was approximately 10 mg/ml. Commercial crystallization screen solutions were dispensed into 96-well plates using an Art-Robbins Instruments Griffon dispensing robot (Alpha Biotech Ltd.) in sitting drop vapor diffusion format. Drops containing 200 nl of screen solution and 200 nl of protein solution were equilibrated against a reservoir of 60 μl of crystallization solution. The plates were sealed and incubated at 18°C.

Crystals of HAdV-D26K appeared in PACT Premier conditions B01 and B04 [0.1 M MIB (malonic acid, imidazole, and boric acid) and 25% w/v polyethylene glycol 1500, pH 4.0 and pH 8.0, respectively], within 1 to 7 days. Crystals were then soaked in reservoir solution containing Neu5Ac (Sigma-Aldrich, cat. no. A2388) at a final concentration of 10 mM and incubated overnight before harvest. Crystals were cryoprotected with reservoir solution to which ethylene glycol was added at a final concentration of 25%. Crystals were harvested in thin plastic loops and stored in liquid nitrogen for transfer to the synchrotron. Data were collected at Diamond Light Source beamline I04, running at a wavelength of 0.9795 Å. During data collection, crystals were maintained in a cold air stream at 100°K. Dectris PILATUS 6M detectors recorded the diffraction patterns, which were analyzed and reduced with XDS (*55*), Xia2, DIALS (*56*), and autoProc (*57*). Scaling and merging data were completed with Pointless, Aimless and Truncate from the CCP4 package (*58*). Structures were solved with Phaser, COOT was used to correct the sequences and adjust the models, and REFMAC5 was used to refine the structures and calculate maps. Graphical representations were prepared with PyMOL. Reflection data and final models were deposited in the PDB database with accession codes 6QU6, 6QU8, and 6FJO. Full crystallographic refinement statistics are given in table S1.

### Calculation of electrostatic surface potentials and pIs

HAdV-D37, HAdV-D19p, and HAdV-D26 used PDB IUXA (*37*), PDB 1UXB (*37*), and PDB 6QU8, respectively, as the input. HAdV-D8 was calculated using a homology model, generated as described below, for input.

The PDB2PQR server (V 2.1.1) (http://nbcr-222.ucsd.edu/pdb2pqr_2.1.1/) was used to assign charge and radius parameters using the PARSE force field and assign protonation states using Propka, at pH 7.35. APBS was used to calculate electrostatic surface potentials, and the map output was visualized in PyMOL (*59*).

### Homology modeling of adenovirus type 8

The I-TASSER protein structure and function prediction server (https://zhanglab.ccmb.med.umich.edu/I-TASSER/) (*60*) was used to generate a homology model of HAdV-D8 based on the published sequence of HAdV-D8 (*22*), using the published structure of its closest relative (by sequence identity), HAdV-D19p (*37*). The resultant monomer was then copied three times, using the HAdV-19p trimer as a template and the monomers aligned in PyMOL to generate a model of the complete HAdV-D8K trimer.

## Supplementary Material

http://advances.sciencemag.org/cgi/content/full/5/9/eaax3567/DC1

Download PDF

Human adenovirus type 26 uses sialic acid–bearing glycans as a primary cell entry receptor

## SUPPLEMENTARY MATERIALS

Supplementary material for this article is available at http://advances.sciencemag.org/cgi/content/full/5/9/eaax3567/DC1 Fig. S1. Sialic acid forms a stable interaction with HAdV-D26K 654 at both pH 4.0 (PDB 6QU6) and pH 8.0 (PDB 6QU8). Fig. S2. Structure of sialic acid (Neu5Ac) in a biologically relevant conformation. Fig. S3. HAdV-D26K forms a similar interaction with sialic acid at both pH 4.0 (PDB 6QU6) and pH 8.0 (PDB 6QU8) through a combination of polar, water bridge, and hydrophobic interactions. Fig. S4. Species D adenoviruses conserve known sialic acid–binding residues. Table S1. Data collection and refinement statistics for structures generated in this study. Table S2. Primers used to generate recombineering PCR products in this study.

## References

[1] Lion T., Adenovirus infections in immunocompetent and immunocompromised patients. Clin. Microbiol. Rev. 27, 441–462 (2014). 24982316 10.1128/CMR.00116-13 PMC4135893

[2] Garcia-Zalisnak D., Rapuano C., Sheppard J. D., Davis A. R., Adenovirus ocular infections: Prevalence, pathology, pitfalls, and practical pointers. Eye Contact Lens 44 ( suppl. 1), S1–S7 (2018). 10.1097/ICL.0000000000000226 29664772

[3] Civljak R., tot T., Falsey A. R., Huljev E., Vranes J., Ljubin-Sternak S., Viral pathogens associated with acute respiratory illness in hospitalized adults and elderly from Zagreb, Croatia, 2016 to 2018. J. Med. Virol. 91, 1202–1209 (2019). 30801727 10.1002/jmv.25437 PMC7166480

[4] Robinson C. M., Singh G., Henquell C., Walsh M. P., Peigue-Lafeuille H., Seto D., Jones M. S., Dyer D. W., Chodosh J., Computational analysis and identification of an emergent human adenovirus pathogen implicated in a respiratory fatality. Virology 409, 141–147 (2011). 21056888 10.1016/j.virol.2010.10.020 PMC3006489

[5] Killerby M. E., Rozwadowski F., Lu X., Caulcrick-Grimes M., McHugh L., Haldeman A. M., Fulton T., Schneider E., Sakthivel S. K., Bhatnagar J., Rabeneck D. B., Zaki S., Gerber S. I., Watson J. T., Respiratory illness associated with emergent human adenovirus genome type 7d, New Jersey, 2016–2017. Open Forum Infect. Dis. 6, ofz017 (2019). 30800698 10.1093/ofid/ofz017 PMC6379021

[6] Potter R. N., Cantrell J. A., Mallak C. T., Gaydos J. C., Adenovirus-associated deaths in US military during postvaccination period, 1999–2010. Emerg. Infect. Dis. 18, 507–509 (2012). 22377242 10.3201/eid1803.111238 PMC3309579

[7] Khanal S., Ghimire P., Dhamoon A. S., The repertoire of adenovirus in human disease: The innocuous to the deadly. Biomedicine 6, E30 (2018). 10.3390/biomedicines6010030 PMC5874687 29518985

[8] Norris S. H., Butler T. C., Glass N., Tran R., Fatal hepatic necrosis caused by disseminated type 5 adenovirus infection in a renal transplant recipient. Am. J. Nephrol. 9, 101–105 (1989). 2545099 10.1159/000167945

[9] Al Qurashi Y. M. A., Alkhalaf M. A., Lim L., Guiver M., Cooper R. J., Sequencing and phylogenetic analysis of the hexon, fiber, and penton regions of adenoviruses isolated from AIDS patients. J. Med. Virol. 84, 1157–1165 (2012). 22711343 10.1002/jmv.23331

[10] Hierholzer J. C., Adenoviruses in the immunocompromised host. Clin. Microbiol. Rev. 5, 262–274 (1992). 1323383 10.1128/cmr.5.3.262 PMC358244

[11] International Committee on Taxonomy of Viruses, *Virus Taxonomy: Classification and Nomenclature of Viruses: Sixth Report of the International Committee on Taxonomy of Viruses* (Springer-Verlag, Wien, New York, 1995), Archives of virology. Supplementum; 10.

[12] HAdV Working Group, http://hadvwg.gmu.edu/.

[13] Seiradake E., Lortat-Jacob H., Billet O., Kremer E. J., Cusack S., Structural and mutational analysis of human Ad37 and canine adenovirus 2 fiber heads in complex with the D1 domain of coxsackie and adenovirus receptor. J. Biol. Chem. 281, 33704–33716 (2006). 16923808 10.1074/jbc.M605316200

[14] Gaggar A., Shayakhmetov D. M., Lieber A., CD46 is a cellular receptor for group B adenoviruses. Nat. Med. 9, 1408–1412 (2003). 14566335 10.1038/nm952

[15] Vassal-Stermann E., Effantin G., Zubieta C., Burmeister W., Iseni F., Wang H., Lieber A., Schoehn G., Fender P., CryoEM structure of adenovirus type 3 fibre with desmoglein 2 shows an unusual mode of receptor engagement. Nat. Commun. 10, 1181 (2019). 30862836 10.1038/s41467-019-09220-y PMC6414520

[16] Arnberg N., Edlund K., Kidd A. H., Wadell G., Adenovirus type 37 uses sialic acid as a cellular receptor. J. Virol. 74, 42–48 (2000). 10590089 PMC111511

[17] Abbink P., Lemckert A. A. C., Ewald B. A., Lynch D. M., Denholtz M., Smits S., Holterman L., Damen I., Vogels R., Thorner A. R., O’Brien K. L., Carville A., Mansfield K. G., Goudsmit J., Havenga M. J. E., Barouch D. H., Comparative seroprevalence and immunogenicity of six rare serotype recombinant adenovirus vaccine vectors from subgroups B and D. J. Virol. 81, 4654–4663 (2007). 17329340 10.1128/JVI.02696-06 PMC1900173

[18] Pauly M., Hoppe E., Mugisha L., Petrzelkova K., Akoua-Koffi C., Couacy-Hymann E., Anoh A., Mossoun A., Schubert G., Wiersma L., Pascale S., Muyembe J. J., Karhemere S., Weiss S., Leendertz S., Calvignac-Spencer S., Leendertz F. H., Ehlers B., High prevalence and diversity of species D adenoviruses (HAdV-D) in human populations of four Sub-Saharan countries. Virol. J. 11, 25 (2014). 24512686 10.1186/1743-422X-11-25 PMC3928611

[19] Dakin R. S., Parker A. L., Delles C., Nicklin S. A., Baker A. H., Efficient transduction of primary vascular cells by the rare adenovirus serotype 49 vector. Hum. Gene Ther. 26, 312–319 (2015). 25760682 10.1089/hum.2015.019 PMC4442572

[20] Swenson P. D., Lowens M. S., Celum C. L., Hierholzer J. C., Adenovirus types 2, 8, and 37 associated with genital infections in patients attending a sexually transmitted disease clinic. J. Clin. Microbiol. 33, 2728–2731 (1995). 8567914 10.1128/jcm.33.10.2728-2731.1995 PMC228564

[21] Aoki K., Tagawa Y., A twenty-one year surveillance of adenoviral conjunctivitis in Sapporo, Japan. Int. Ophthalmol. Clin. 42, 49–54 (2002). 12189615 10.1097/00004397-200201000-00008

[22] Kaneko H., Iida T., Ishiko H., Ohguchi T., Ariga T., Tagawa Y., Aoki K., Ohno S., Suzutani T., Analysis of the complete genome sequence of epidemic keratoconjunctivitis-related human adenovirus type 8, 19, 37 and a novel serotype. J. Gen. Virol. 90, 1471–1476 (2009). 19264666 10.1099/vir.0.009225-0

[23] Zhou X., Robinson C. M., Rajaiya J., Dehghan S., Seto D., Jones M. S., Dyer D. W., Chodosh J., Analysis of human adenovirus type 19 associated with epidemic keratoconjunctivitis and its reclassification as adenovirus type 64. Invest. Ophthalmol. Vis. Sci. 53, 2804–2811 (2012). 22467570 10.1167/iovs.12-9656 PMC3367469

[24] Walsh M. P., Chintakuntlawar A., Robinson C. M., Madisch I., Harrach B., Hudson N. R., Schnurr D., Heim A., Chodosh J., Seto D., Jones M. S., Evidence of molecular evolution driven by recombination events influencing tropism in a novel human adenovirus that causes epidemic keratoconjunctivitis. PLOS ONE 4, e5635 (2009). 19492050 10.1371/journal.pone.0005635 PMC2685984

[25] Aoki K., Kitaichi N., Hinokuma R., Ohno S., Characteristics of epidemic keratoconjunctivitis due to novel adenovirus types. J. Clin. Ophthalmol. Eye Disord. 1, 1013 (2017).

[26] Matsuura K., Terasaka Y., Uchio E., Saeki Y., Fujimoto T., Hanaoka N., Miyazaki D., Inoue Y., Human adenoviral type 54 keratoconjunctivitis accompanied by stellate keratitis and keratic precipitates: Two cases. BMC Ophthalmol. 19, 7 (2019). 30616635 10.1186/s12886-018-1025-6 PMC6323660

[27] Huang G., Yao W., Yu W., Mao L., Sun H., Yao W., Tian J., Wang L., Bo Z., Zhu Z., Zhang Y., Zhao Z., Xu W., Outbreak of epidemic keratoconjunctivitis caused by human adenovirus type 56, China, 2012. PLOS ONE 9, e110781 (2014). 25343525 10.1371/journal.pone.0110781 PMC4208770

[28] Stanton R. J., McSharry B. P., Armstrong M., Tomasec P., Wilkinson G. W. G., Re-engineering adenovirus vector systems to enable high-throughput analyses of gene function. Biotechniques 45, 659, 664–662, 668 (2008). 19238796 10.2144/000112993

[29] Uusi-Kerttula H., Hulin-Curtis S., Davies J., Parker A. L., Oncolytic adenovirus: Strategies and insights for vector design and immuno-oncolytic applications. Viruses 7, 6009–6042 (2015). 26610547 10.3390/v7112923 PMC4664994

[30] Majhen D., Calderon H., Chandra N., Fajardo C. A., Rajan A., Alemany R., Custers J., Adenovirus-based vaccines for fighting infectious diseases and cancer: Progress in the field. Hum. Gene Ther. 25, 301–317 (2014). 24580050 10.1089/hum.2013.235

[31] Barouch D. H., Tomaka F. L., Wegmann F., Stieh D. J., Alter G., Robb M. L., Michael N. L., Peter L., Nkolola J. P., Borducchi E. N., Chandrashekar A., Jetton D., Stephenson K. E., Li W., Korber B., Tomaras G. D., Montefiori D. C., Gray G., Frahm N., McElrath M. J., Baden L., Johnson J., Hutter J., Swann E., Karita E., Kibuuka H., Mpendo J., Garrett N., Mngadi K., Chinyenze K., Priddy F., Lazarus E., Laher F., Nitayapan S., Pitisuttithum P., Bart S., Campbell T., Feldman R., Lucksinger G., Borremans C., Callewaert K., Roten R., Sadoff J., Scheppler L., Weijtens M., Feddes-de Boer K., van Manen D., Vreugdenhil J., Zahn R., Lavreys L., Nijs S., Tolboom J., Hendriks J., Euler Z., Pau M. G., Schuitemaker H., Evaluation of a mosaic HIV-1 vaccine in a multicentre, randomised, double-blind, placebo-controlled, phase 1/2a clinical trial (APPROACH) and in rhesus monkeys (NHP 13-19). Lancet 392, 232–243 (2018). 30047376 10.1016/S0140-6736(18)31364-3 PMC6192527

[32] Cox F., van der Fits L., Abbink P., Larocca R. A., van Huizen E., Saeland E., Verhagen J., Peterson R., Tolboom J., Kaufmann B., Schuitemaker H., Barouch D. H., Zahn R., Adenoviral vector type 26 encoding Zika virus (ZIKV) M-Env antigen induces humoral and cellular immune responses and protects mice and nonhuman primates against ZIKV challenge. PLOS ONE 13, e0202820 (2018). 30142207 10.1371/journal.pone.0202820 PMC6108497

[33] Long-term Safety Follow-up of Participants Exposed to the Candidate Ebola Vaccines Ad26.ZEBOV and/or MVA-BN-Filo—Full Text View—ClinicalTrials.gov, https://clinicaltrials.gov/ct2/show/NCT02661464?term=adenovirus&recrs=a&cond=ebola&rank=1.

[34] Trial of the Safety and Immunogenicity of an Oral, Replicating Ad26 Vectored HIV-1 Vaccine—Full Text View—ClinicalTrials.gov, https://clinicaltrials.gov/ct2/show/NCT02366013.

[35] A Shedding Study of Adenovirus Serotype 26 Based Respiratory Syncytial Virus Pre-fusion F Protein (Ad26.RSV.preF) Vaccine in Adults—Full Text View—ClinicalTrials.gov, https://clinicaltrials.gov/ct2/show/NCT03795441.

[36] Baker A. T., Greenshields-Watson A., Coughlan L., Davies J. A., Uusi-Kerttula H., Cole D. K., Rizkallah P. J., Parker A. L., Diversity within the adenovirus fiber knob hypervariable loops influences primary receptor interactions. Nat. Commun. 10, 741 (2019). 30765704 10.1038/s41467-019-08599-y PMC6376029

[37] Burmeister W. P., Guilligay D., Cusack S., Wadell G., Arnberg N., Crystal structure of species D adenovirus fiber knobs and their sialic acid binding sites. J. Virol. 78, 7727–7736 (2004). 15220447 10.1128/JVI.78.14.7727-7736.2004 PMC434083

[38] Arnberg N., Pring-Åkerblom P., Wadell G., Adenovirus type 37 uses sialic acid as a cellular receptor on Chang C cells. J. Virol. 76, 8834–8841 (2002). 12163603 10.1128/JVI.76.17.8834-8841.2002 PMC136979

[39] Arcasoy S. M., Latoche J., Gondor M., Watkins S. C., Henderson R. A., Hughey R., Finn O. J., Pilewski J. M., MUC1 and other sialoglycoconjugates inhibit adenovirus-mediated gene transfer to epithelial cells. Am. J. Respir. Cell Mol. Biol. 17, 422–435 (1997). 9376117 10.1165/ajrcmb.17.4.2714

[40] Chandra N., Frängsmyr L., Imhof S., Caraballo R., Elofsson M., Arnberg N., Sialic acid-containing glycans as cellular receptors for ocular human adenoviruses: Implications for tropism and treatment. Viruses 11, E395 (2019). 31035532 10.3390/v11050395 PMC6563162

[41] Nilsson E. C., Storm R. J., Bauer J., Johansson S. M. C., Lookene A., Ångström J., Hedenström M., Eriksson T. L., Frängsmyr L., Rinaldi S., Willison H. J., Domellöf F. P., Stehle T., Arnberg N., The GD1a glycan is a cellular receptor for adenoviruses causing epidemic keratoconjunctivitis. Nat. Med. 17, 105–109 (2011). 21151139 10.1038/nm.2267

[42] Seiradake E., Henaff D., Wodrich H., Billet O., Perreau M., Hippert C., Mennechet F., Schoehn G., Lortat-Jacob H., Dreja H., Ibanes S., Kalatzis V., Wang J. P., Finberg R. W., Cusack S., Kremer E. J., The cell adhesion molecule “CAR” and sialic acid on human erythrocytes influence adenovirus in vivo biodistribution. PLOS Pathog. 5, e1000277 (2009). 19119424 10.1371/journal.ppat.1000277 PMC2607015

[43] Singh A. K., Berbís M. Á., Ballmann M. Z., Kilcoyne M., Menéndez M., Nguyen T. H., Joshi L., Cañada F. J., Jiménez-Barbero J., Benkő M., Harrach B., van Raaij M. J., Structure and sialyllactose binding of the carboxy-terminal head domain of the fibre from a siadenovirus, turkey adenovirus 3. PLOS ONE 10, e0139339 (2015). 26418008 10.1371/journal.pone.0139339 PMC4587935

[44] Lenman A., Liaci A. M., Liu Y., Årdahl C., Rajan A., Nilsson E., Bradford W., Kaeshammer L., Jones M. S., Frängsmyr L., Feizi T., Stehle T., Arnberg N., Human adenovirus 52 uses sialic acid-containing glycoproteins and the coxsackie and adenovirus receptor for binding to target cells. PLOS Pathog. 11, e1004657 (2015). 25674795 10.1371/journal.ppat.1004657 PMC4335501

[45] Amoureux M.-C., Coulibaly B., Chinot O., Loundou A., Metellus P., Rougon G., Figarella-Branger D., Polysialic acid neural cell adhesion molecule (PSA-NCAM) is an adverse prognosis factor in glioblastoma, and regulates olig2 expression in glioma cell lines. BMC Cancer 10, 91 (2010). 20219118 10.1186/1471-2407-10-91 PMC2854115

[46] Arnberg N., Kidd A. H., Edlund K., Olfat F., Wadell G., Initial interactions of subgenus D adenoviruses with A549 cellular receptors: Sialic acid versus α_v_integrins. J. Virol. 74, 7691–7693 (2000). 10906228 10.1128/jvi.74.16.7691-7693.2000 PMC112295

[47] Lenman A., Liaci A. M., Liu Y., Frängsmyr L., Frank M., Blaum B. S., Chai W., Podgorski I. I., Harrach B., Benkő M., Feizi T., Stehle T., Arnberg N., Polysialic acid is a cellular receptor for human adenovirus 52. Proc. Natl. Acad. Sci. U.S.A. 115, E4264–E4273 (2018). 29674446 10.1073/pnas.1716900115 PMC5939068

[48] Huang S., Reddy V., Dasgupta N., Nemerow G. R., A single amino acid in the adenovirus type 37 fiber confers binding to human conjunctival cells. J. Virol. 73, 2798–2802 (1999). 10074127 10.1128/jvi.73.4.2798-2802.1999 PMC104037

[49] Nestić D., Uil T. G., Ma J., Roy S., Vellinga J., Baker A. H., Custers J., Majhen D., αvβ3 integrin is required for efficient infection of epithelial cells with human adenovirus type 26. J. Virol. 93, e01474-18 (2019). 30333171 10.1128/JVI.01474-18 PMC6288336

[50] Yu X., Veesler D., Campbell M. G., Barry M. E., Asturias F. J., Barry M. A., Reddy V. S., Cryo-EM structure of human adenovirus D26 reveals the conservation of structural organization among human adenoviruses. Sci. Adv. 3, e1602670 (2017). 28508067 10.1126/sciadv.1602670 PMC5425241

[51] Spjut S., Qian W., Bauer J., Storm R., Frängsmyr L., Stehle T., Arnberg N., Elofsson M., A potent trivalent sialic acid inhibitor of adenovirus type 37 infection of human corneal cells. Angew. Chem. Int. Ed. Engl. 50, 6519–6521 (2011). 21648036 10.1002/anie.201101559 PMC3210828

[52] Uusi-Kerttula H., Legut M., Davies J., Jones R., Hudson E., Hanna L., Stanton R. J., Chester J. D., Parker A. L., Incorporation of peptides targeting EGFR and FGFR1 into the adenoviral fiber knob domain and their evaluation as targeted cancer therapies. Hum. Gene Ther. 26, 320–329 (2015). 25919378 10.1089/hum.2015.015 PMC4442602

[53] Sievers F., Wilm A., Dineen D., Gibson T. J., Karplus K., Li W., Lopez R., McWilliam H., Remmert M., Söding J., Thompson J. D., Higgins D. G., Fast, scalable generation of high-quality protein multiple sequence alignments using Clustal Omega. Mol. Syst. Biol. 7, 539 (2011). 21988835 10.1038/msb.2011.75 PMC3261699

[54] Uusi-Kerttula H., Davies J., Coughlan L., Hulin-Curtis S., Jones R., Hanna L., Chester J. D., Parker A. L., Pseudotyped αvβ6 integrin-targeted adenovirus vectors for ovarian cancer therapies. Oncotarget 7, 27926–27937 (2016). 27056886 10.18632/oncotarget.8545 PMC5053699

[55] Kabsch W., XDS. Acta Crystallogr. D Biol. Crystallogr. 66, 125–132 (2010). 20124692 10.1107/S0907444909047337 PMC2815665

[56] Winter G., Waterman D. G., Parkhurst J. M., Brewster A. S., Gildea R. J., Gerstel M., Fuentes-Montero L., Vollmar M., Michels-Clark T., Young I. D., Sauter N. K., Evans G., DIALS: Implementation and evaluation of a new integration package. Acta Crystallogr. D Struct. Biol. 74, 85–97 (2018). 29533234 10.1107/S2059798317017235 PMC5947772

[57] Vonrhein C., Flensburg C., Keller P., Sharff A., Smart O., Paciorek W., Womack T., Bricogne G., Data processing and analysis with the *autoPROC* toolbox. Acta Crystallogr. D Biol. Crystallogr. 67, 293–302 (2011). 21460447 10.1107/S0907444911007773 PMC3069744

[58] Dodson E. J., Winn M., Ralph A., Collaborative computational project, number 4: Providing programs for protein crystallography. Methods Enzymol. 277, 620–633 (1997). 18488327 10.1016/s0076-6879(97)77034-4

[59] Dolinsky T. J., Nielsen J. E., McCammon J. A., Baker N. A., PDB2PQR: An automated pipeline for the setup of Poisson–Boltzmann electrostatics calculations. Nucleic Acids Res. 32, W665–W667 (2004). 15215472 10.1093/nar/gkh381 PMC441519

[60] Yang J., Yan R., Roy A., Xu D., Poisson J., Zhang Y., The I-TASSER Suite: Protein structure and function prediction. Nat. Methods 12, 7–8 (2015). 25549265 10.1038/nmeth.3213 PMC4428668

