# Supplementary material for: Human adenovirus type 26 uses sialic acid–bearing glycans as a primary cell entry receptor
Source: Sci Adv. 2019 Sep 4;5(9):eaax3567. doi: 10.1126/sciadv.aax3567 (PMC6726447; doi:10.1126/sciadv.aax3567)
Supplement: http://advances.sciencemag.org/cgi/content/full/5/9/eaax3567/DC1 [file supp_5_9_eaax3567__index.html]

Science Advances | Science AdvancesAAASSearchScience AdvancesMenu

## Supplementary Materials

**This PDF file includes:**

- Fig. S1. Sialic acid forms a stable interaction with HAdV-D26K 654 at both pH 4.0 (PDB 6QU6) and pH 8.0 (PDB 6QU8).
- Fig. S2. Structure of sialic acid (Neu5Ac) in a biologically relevant conformation.
- Fig. S3. HAdV-D26K forms a similar interaction with sialic acid at both pH 4.0 (PDB 6QU6) and pH 8.0 (PDB 6QU8) through a combination of polar, water bridge, and hydrophobic interactions.
- Fig. S4. Species D adenoviruses conserve known sialic acid–binding residues.
- Table S1. Data collection and refinement statistics for structures generated in this study.
- Table S2. Primers used to generate recombineering PCR products in this study.

Download PDF

**Files in this Data Supplement:**

- Adobe PDF - aax3567\_SM.pdf
