# Supplementary material for: Human adenovirus type 26 uses sialic acid–bearing glycans as a primary cell entry receptor
Source: Sci Adv. 2019 Sep 4;5(9):eaax3567. doi: 10.1126/sciadv.aax3567 (PMC6726447; doi:10.1126/sciadv.aax3567)
Supplement: Download PDF [file aax3567_SM.pdf]

Science Advances
AAAS

advances.sciencemag.org/cgi/content/full/5/9/eaax3567/DC1

Supplementary Materials for

Human adenovirus type 26 uses sialic acid–bearing glycans as a primary cell entry receptor

Alexander T. Baker, Rosie M. Mundy, James A. Davies, Pierre J. Rizkallah, Alan L. Parker*

*Corresponding author. Email: parkeral@cardiff.ac.uk

Published 4 September 2019, *Sci. Adv.* **5**, eaax3567 (2019)
DOI: 10.1126/sciadv.aax3567

This PDF file includes:

Fig. S1. Sialic acid forms a stable interaction with HAdV-D26K 654 at both pH 4.0 (PDB 6QU6) and pH 8.0 (PDB 6QU8).
Fig. S2. Structure of sialic acid (Neu5Ac) in a biologically relevant conformation.
Fig. S3. HAdV-D26K forms a similar interaction with sialic acid at both pH 4.0 (PDB 6QU6) and pH 8.0 (PDB 6QU8) through a combination of polar, water bridge, and hydrophobic interactions.
Fig. S4. Species D adenoviruses conserve known sialic acid–binding residues.
Table S1. Data collection and refinement statistics for structures generated in this study.
Table S2. Primers used to generate recombineering PCR products in this study.

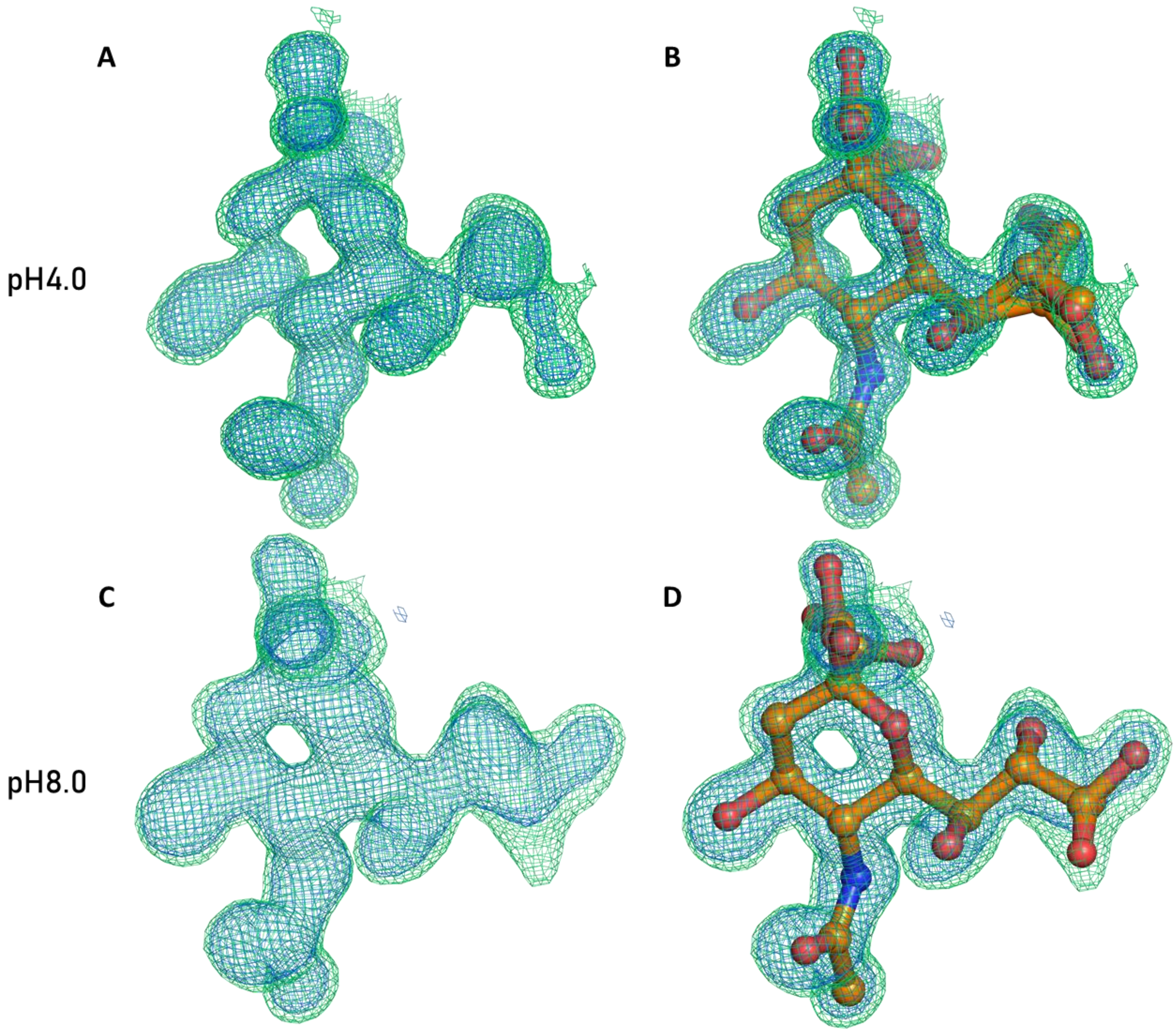

Fig. S1. Sialic acid forms a stable interaction with HAdV-D26K 654 at both pH 4.0 (PDB 6QU6) and pH 8.0 (PDB 6QU8). The omit map at pH4.0 (**A**) shows density for a small molecule ligand, which can be best modelled by a sialic acid double conformer (**B**). The same is true at pH8.0 (**C**), but the preferred conformations of the glycerol group are different (**D**). Crystallisation statistics in supplementary table 1, 2FoFc map (blue mesh, σ=1.5), FoFc (Green mesh, σ=3.0).

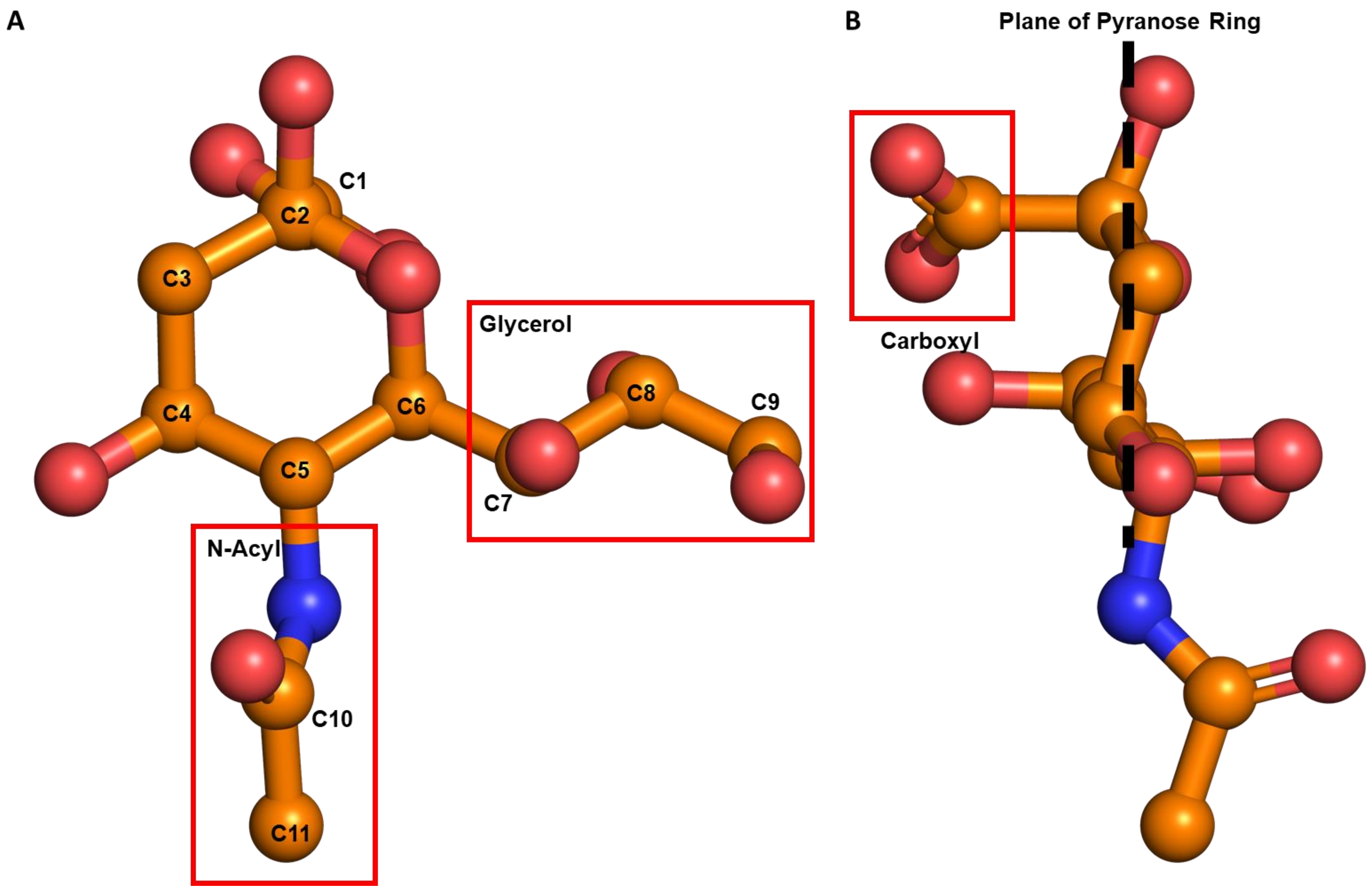

Fig. S2. Structure of sialic acid (Neu5Ac) in a biologically relevant conformation. Viewing the Neu5Ac face on with glycerol and N-Acetyl groups labelled in red boxes, and the carbons numbered (**A**). Side on the carboxyl group (red box) is seen in the axial conformation with the C2 OH group planar to the ring (**B**).

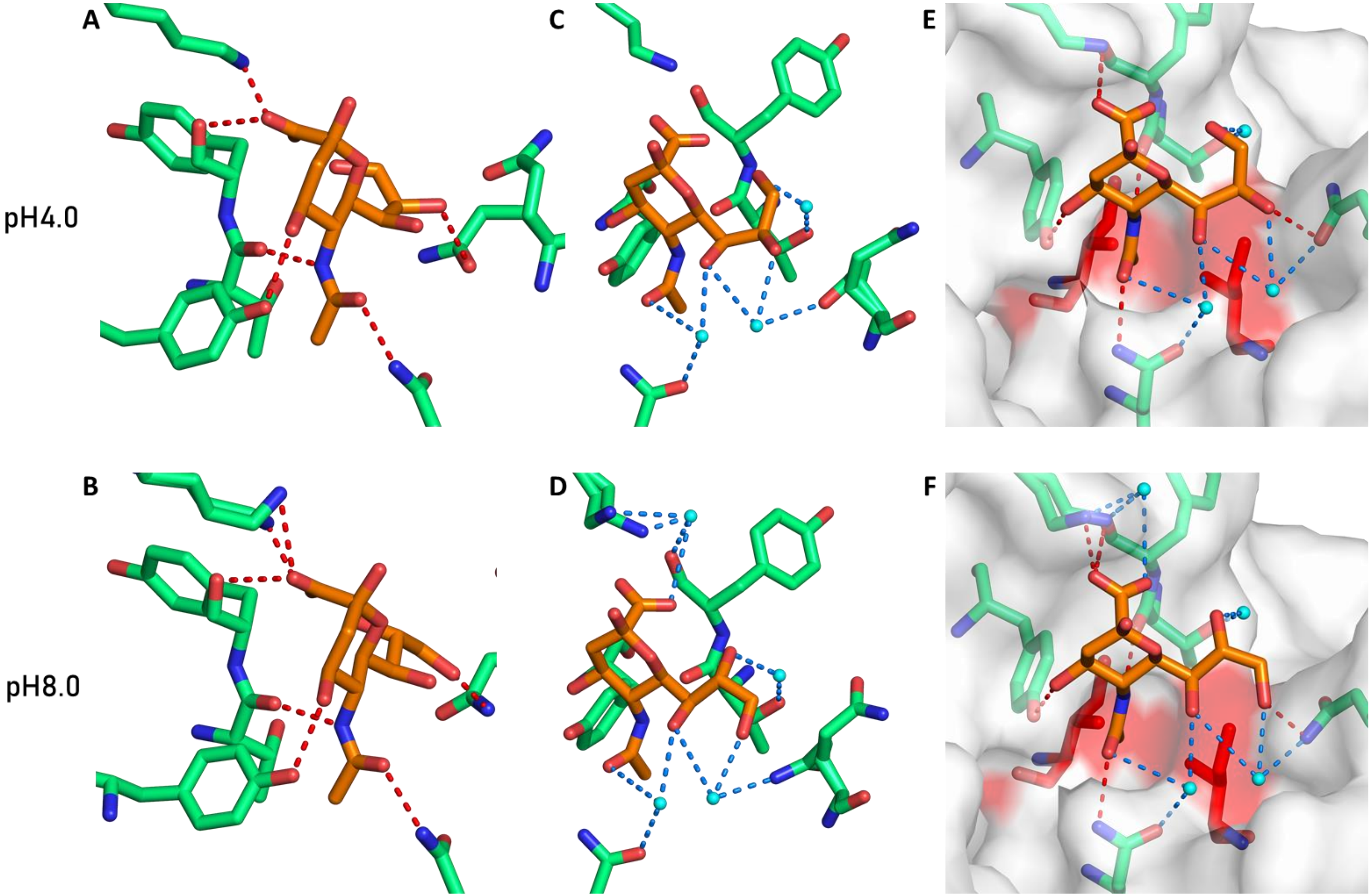

Fig. S3. HAdV-D26K forms a similar interaction with sialic acid at both pH 4.0 (PDB 6QU6) and pH 8.0 (PDB 6QU8) through a combination of polar, water bridge, and hydrophobic interactions. At pH4.0 sialic acid forms numerous polar contacts to charged side chains in HAdV-D26K (**A**), similar contacts are seen at pH8.0 and exhibits a lysine double conformer (**B**). At pH4.0 sialic acid forms several water bridges stabilising the interaction of the glycerol group (**C**), the same bridges are seen at pH8.0 with the addition of a water-bridge contact on the carboxyl group not seen at pH4.0 (**D**). A hydrophobic interface is formed around the N-Acetyl methyl group which appears to be similar and stable at pH4.0 (**E**) and pH8.0 (**F**). Polar bonds to residues are shown as red dashes, water-bridge contacts as blue dashes, and waters as cyan spheres. Sialic acid is shown in orange, polar HAdV-D26K residues as green sticks, and purely hydrophobic residues as red sticks. The HAdV-D26 surface is shown in white with hydrophobic regions in red. Oxygen and nitrogen atoms are coloured red and blue, respectively.

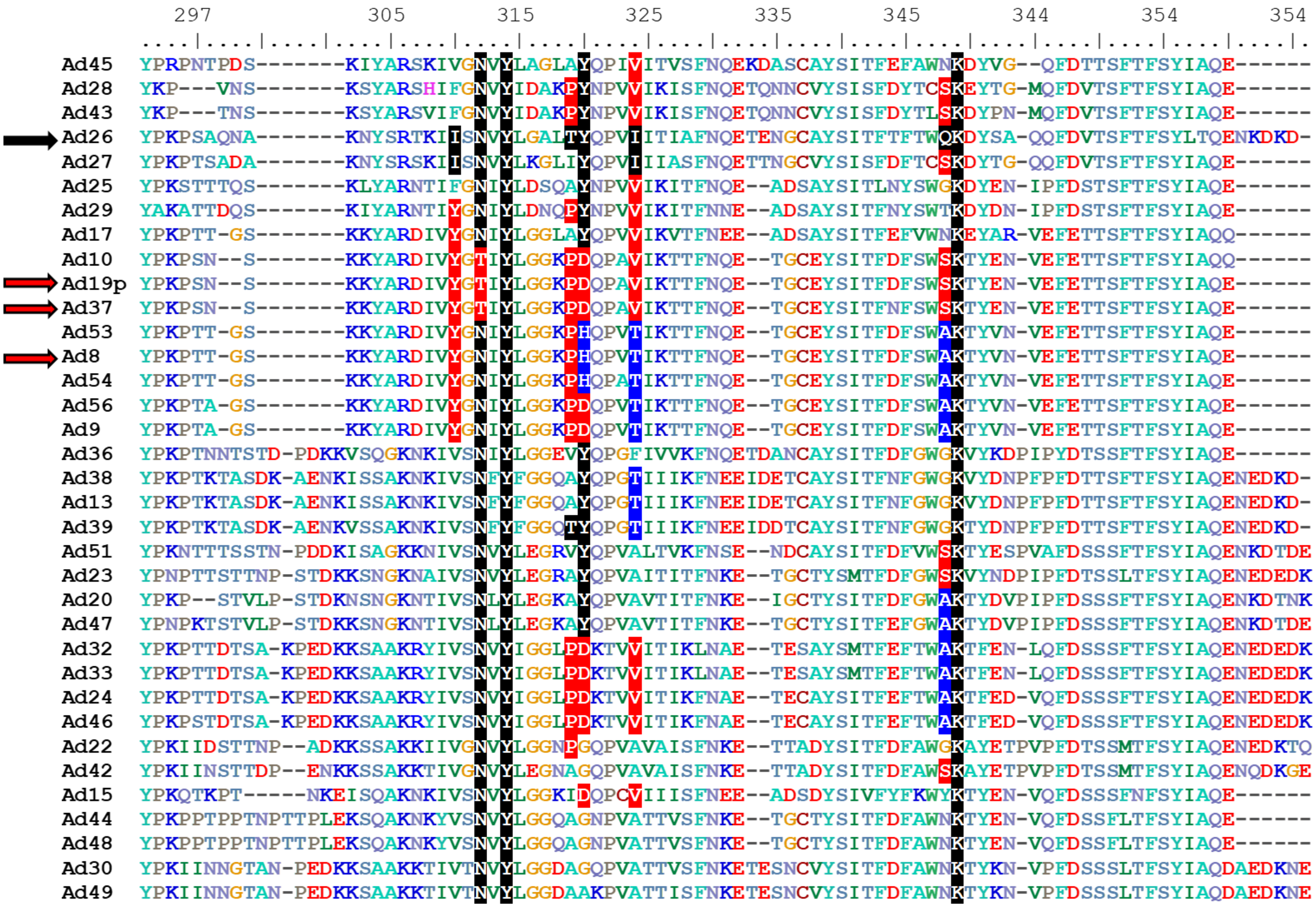

Fig. S4. Species D adenoviruses conserve known sialic acid–binding residues. Sequence alignment of species D adenovirus fiber-knob proteins. Known HAdV-D26 and HAdV-D37/64/19p residues forming contacts with sialic acid are highlighted in black and red respectively. Homologous residues are coloured similarly to the virus which they share the residue with. HAdV-D8 residues at known sialic acid binding locations which are dissimilar to HAdV-D26/37 are highlighted in blue, as are homologous residues in other viruses. Names utilise the short nomenclature, all are human species D adenoviruses. Numbering is for HAdV-D26K.

Table S1. Data collection and refinement statistics for structures generated in this study.

PDB Entry	6QU6	6QU8	6FJO
**Data Collection**			
Diamond Beamline	I04	I04	I04
Date	25/10/2018	25/10/2018	05/12/2017
Wavelength	0.91587	0.91587	0.9795
**Crystal Data**			
Crystallisation Conditions	0.1 M MIB, 25 % w/v PEG 1500	0.1 M MIB, 25 % w/v PEG 1500	0.1 M SPG, 25% PEG 1500
pH	4.0	8.0	4.0
a=*b*=*c* (Å)	85.73	85.92	85.78
α=β=γ (°)	90.0	90.0	90.0
Space group	$P\ 2_1\ 3$	$P\ 2_1\ 3$	P 21 3
Resolution (Å)	1.03 – 49.5	1.19 – 42.96	1.17-85.78
Outer shell	1.03 – 1.06	1.19 – 1.22	1.17-1.23
R-merge (%)	5.3 (125.3)	8.5 (276.1)	6.5 (137.0)
R-meas (%)	5.5 (161.3)	8.8 (283.2)	6.6 (140.4)
CC1/2	1.0 (0.224)	1.0 (0.505)	1.00 (0.825)
I / σ(I)	21.9 (0.7)	20.7 (1.3)	24.8 (2.3)
Completeness (%)	97.7 (76.5)	100.0 (100.0)	100.0 (100.0)
Multiplicity	14.9 (2.0)	21.4 (20.3)	21.8 (21.2)
Total Measurements	1,509,159	1,448,478	1,568,641
Unique Reflections	103,975	67,799	71,878
Wilson B-factor($Å^2$)	8.4	11.1	12.2
**Refinement Statistics**			
Total number of refined atoms	1,936	1,846	1,761
R-work reflections	96,027	64,286	68,283
R-free reflections	4,907	3,441	3,558
R-work/R-free (%)	13.6 / 14.8	14.17 / 17.20	17.0 / 19.0
**rms deviations**			
Bond lengths (Å)	0.012	0.011	0.021
Bond Angles (°)	1.754	1.661	2.080
[1]Coordinate error	NULL	NULL	0.026
Mean B value ($Å^2$)	17.6	29.6	19.9
**Ramachandran Statistics**			
Favoured/allowed/Outliers	119 / 9 / 0	126 / 10 / 0	133 / 10 / 1
%	93.0 / 7.0 / 0.0	92.7 / 7.4 / 0.0	92.4 / 6.9 / 0.7

* One crystal was used for determining each structure.
* Figures in brackets refer to outer resolution shell, where applicable.
[1] Coordinate Estimated Standard Uncertainty in (Å), calculated based on maximum likelihood statistics.

Buffers:
- MIB: Malonic acid, Imidazole, Boric acid
- SPG: Succinic acid, Sodium phosphate monobasic monohydrate, Glycine: pH 4.0

Table S2. Primers used to generate recombineering PCR products in this study.

Application	Primer	Primer Direction	Primer Sequence
**Cassette Generation**	SacB	Forward	GTTCCTAAACTAGGAACTGGCCTTAGTTTTGACAGCACAGGTGCCATTACAGTAGGAAACAAAAATAATGATAAGCTACCTGTGACGGAAGATCACTTCG
	SacB	Reverse	TATACTACTGAATGAAAAATGACTTGAAATTTTCTGCAATTGAAAAATAAACACGTTGAAACATAACACAAACGATTCTCTGAGGTTCTTATGGCTCTTG
**Generation of HAdV-C5/D26K Pseudotype DNA**	26K	Forward	CTAGGAACTGGCCTTAGTTTTGACAGCACAGGTGCCATTACAGTAGGAAACAAAAATAATGATAAGCTAACCCTGTGGACAACCCCTGACACATCTCCAA
	26K	Reverse	TGAATGAAAAATGACTTGAAATTTTCTGCAATTGAAAAATAAACACGTTGAAACATAACACAAACGATTCTTAGTCTTTGTCTTTGTTCTCTTGGGTAAG
**Generation of HAdV-C5/B35K Pseudotype DNA**	35K	Forward	GTTCCTAAACTAGGAACTGGCCTTAGTTTTGACAGCACAGGTGCCATTACAGTAGGAAACAAAAATAATGATAAGCTAACCTTATGGACTGGAATAAACCCTC
	35K	Reverse	CTACTGAATGAAAAATGACTTGAAATTTTCTGCAATTGAAAAATAAACACGTTGAAACATAACACAAACGATTCTTTAGTTGTCGTCTTCTGTAATGTAAGAAAAG
